# On the Method of Administering Peruvian Bark in Intermittents

**Published:** 1825-04

**Authors:** T. H. Ridgway

**Affiliations:** Licentiate of the Royal College of Physicians, &c.


ART. V.—*On the Method of administering Peruvian Bark in Intermittents.* By T. H. RIDGWAY, M.D. Licentiate of the Royal College of Physicians, &c.

THE efficacy of a medicine in subduing a disease does not alone depend on its possessing powers adapted to this purpose, but also on the time and mode of its administration; and, above all, on the proportion in which it is exhibited.

These several circumstances are, in general, determined by long and patient observation; but it has frequently happened that the prudent caution of the medical profession has kept both the proportion of a medicine, and the frequency of its exhibition, far within the limits to which it might be extended with advantage; and the extent to which it may be administered, not only with safety, but with results hardly to have been expected from the effects it had been observed to produce under a more cautious management, has often been determined by an happy accident.

Few medicines have acquired so high and well-merited a reputation, and maintained it so long, as the Peruvian bark, in fevers of an intermittent character. Indeed, where it has been thought to have failed, and other remedies have been attempted to be introduced to supply its place, it may well be believed (so generally and fully are its virtues acknowledged,) that the partial failure has been the consequence of a rudeness and want of skill in the person who administered it, rather than any deficiency in the powers of the medicine.

In fevers of a pure intermittent type, in which the apyrexy has been distinct and perfect,—and even sometimes in remittents, when the remission has been clear and evident,—it has been the custom to administer the Peruvian bark in substance, in considerable doses, repeated as often as the stomach might be able to bear it, and brought as near as possible to the accession of the approaching paroxysm, so that there might be present, and accumulated in the stomach, at that moment, as much as it would retain, generally amounting to an ounce or an ounce and a half; by which means, when the medicine was carefully introduced, the system prepared for, and the state of the disease admitted of its exhibition, it generally succeeded in putting an end to the paroxysm and the diseased state together. But, on the other hand, it not unfrequently happened that, although all circumstances were favourable to the administration of the medicine, yet the frequency of its repetition disgusted the patient, and it was not taken with due regularity, or in the quantity necessary to avert the expected paroxysm; or the stomach turned from it with loathing, and, although it made an effort to receive the successive doses, rejected them together, before they had sufficiently accumulated and produced their effect; and thus the intention of the physician has been completely frustrated.

It is possible that the concentrated preparations of the Peruvian bark may ultimately supersede the medicine in its unprepared and more bulky form; but it may be a long time before this takes place, and there may be physicians who will continue to think that the form of a powder is the most efficacious, as well as most safe, mode of administering this admirable medicine, as it has hitherto retained its superiority over the usual preparations: it may not, therefore, be altogether useless to make known a method of exhibiting it, whereby it will with more certainty be admitted into, and retained in, the stomach. The disgust occasioned by the frequency of its repetition will be obviated, while its efficacy will by no means be diminished, and its influence over the intermittent paroxysm be, perhaps, rendered more decisive and certain.

I remember, in the year 1809, when I was with the second battalion of the Rifle Brigade at Hythe, in Kent, which had lately returned from the expedition to the Scheldt, and was, to a man, sick of remittent and intermittent fever, so that the whole barrack was converted into an hospital; I was then the only medical officer present, and was forced to trust much to an experienced, although not very intelligent, hospital orderly man.* One day, as this man attended me round the regimental hospital, I directed him to give a soldier, who was in expectation of a paroxysm of intermittent fever, an ounce or an ounce and a half of powdered bark, (I forget which,) in the usual manner, before the fit came on. The next day the man appeared blithe and lively. "Well," said I to the attendant, "did this man take his bark yesterday?" "Yes, sir; he took it about half an hour before the fit came on."—"What! all at once?" "Yes, sir: you ordered it to be taken before the fit, and I gave it him."—"So he drank it all at once?" "Yes, sir."—"And did it make him sick?" "No, sir; not at all."—"And had he any fit afterwards?" "No, sir; he had no fit at all."—"And you feel quite well to-day?" "Yes, sir;" said the soldier.—This was not lost on me. I immediately saw the advantage of being enabled to exhibit the medicine with a certainty and decision I had not before known; and opportunities in abundance soon presented themselves, to enable me to assure myself that it might be administered in this manner with the utmost confidence.

Since that period, I have never thought of giving the Peruvian bark in divided doses, to check the paroxysm of an intermittent, but, where I have thought it proper to use this medicine, have uniformly administered it in one full dose, as near as possible to the approaching paroxysm; and I do not remember to have seen it rejected by the stomach in any instance, and believe that it will not be so liable to this accident as in the shape of divided doses, taken with regularity.

I have frequently directed the powder to be taken in the dose

---

* A soldier attached to the regimental hospital, to attend on the sick, and administer to them food and medicine.

of an ounce and a half, but believe that an ounce is a sufficient quantity, in the greater number of instances, to avert the paroxysm; and, when this has been effected, have been contented with the employment of half an ounce taken for some periods, at the expected time of the paroxysm, until every fear of a return has vanished. Yet I am not sure that I was not in the habit of directing the same quantity to be given the second time, as at first; and, when I felt assured that the paroxysm had been really averted, passed to the diminished proportion.

The powder of bark will mix readily with warm water, and it may afterwards be brought down by cold water to a tepid temperature, when it will be most agreeably taken. A small quantity of water is to be preferred, so as merely to make it potable; and the mouth may be afterwards rinsed out with tepid water, and a little swallowed.

When I was at Madrid in 1812, I was myself affected with intermittent fever, and perfectly succeeded in checking it by the bark employed in this manner; and again in the spring of 1813, when on the frontiers of Portugal.

It may be worthy of remark, that once, when I had kept off the paroxysm for several periods by the use of the bark in half-ounce doses, the supply of this medicine happening to fail, I imagined it might be useful to continue the impression by other bitters. I therefore made a strong infusion of gentian, and, before the calculated period, I took a cup of it; which, by its intense bitterness, produced a shuddering, which was followed by a regular paroxysm, although the disease had been so far removed that I scarcely thought of a return, even without the use of medicine; but the bark being again resorted to, the paroxysms did not afterwards appear.

The advantage of being able to exhibit a distasteful medicine in one full dose, instead of several smaller ones repeated at certain intervals, must be immediately obvious, even supposing that in both ways it has an equally good effect; but, by this mode of administering the Peruvian bark, it is probable that the virtues of the medicine may be brought to bear more effectually on the intermittent paroxysm, and this disagreeable and injurious state be thereby, with more certainty, averted.

*London; February,* 1825.

